# P-888. Impact of Removing ‘Budding Yeast’ From Urinalysis Reports on Fluconazole Use

**DOI:** 10.1093/ofid/ofaf695.1096

**Published:** 2026-01-11

**Authors:** Jill Foster, Christopher M Bland, Bruce M Jones

**Affiliations:** University of Georgia, Savannah, Georgia; University of Georgia College of Pharmacy, Savannah, GA; St. Joseph's/Candler Health System, Savannah, GA

## Abstract

**Background:**

Fluconazole is common in the inpatient setting for treatment of fungal infections. Upon review of data submitted to the National Healthcare Safety Network and calculation of the Standardized Antimicrobial Administration Ratio, our non-profit community health system found opportunity for potential improvement in fluconazole use. One main area of overuse was funguria, especially in asymptomatic patients, when budding yeast was reported on urinalysis. To address this concern, a nudge-based intervention was performed to remove the term "budding yeast" from urinalysis in our 2 hospital health system. This study sought to compare prescribing patterns pre- and post-intervention.

**Figure 1:**
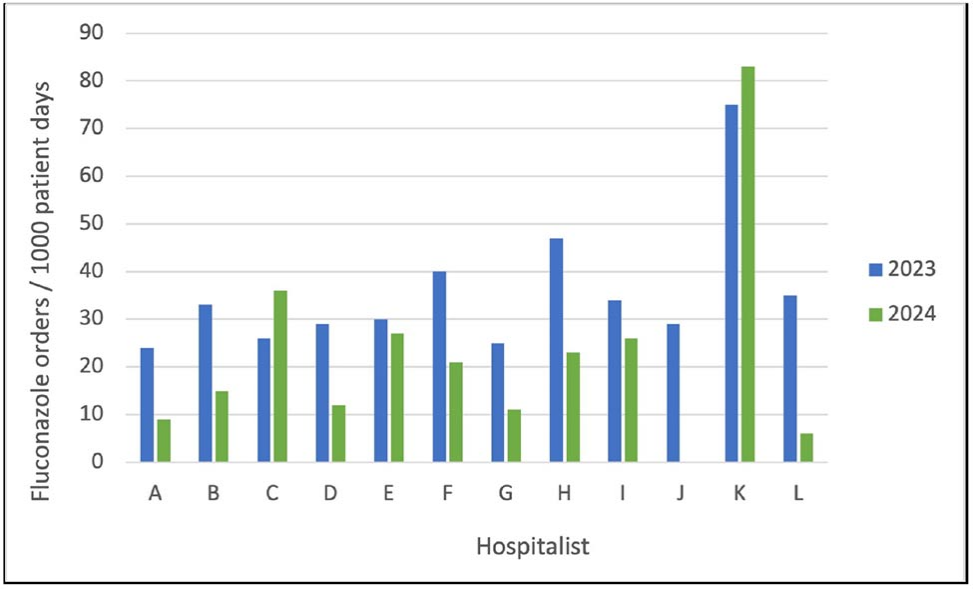
Hospital A - Hospitalist Fluconazole Days of Therapy/1000 Patient Days

**Figure 2:**
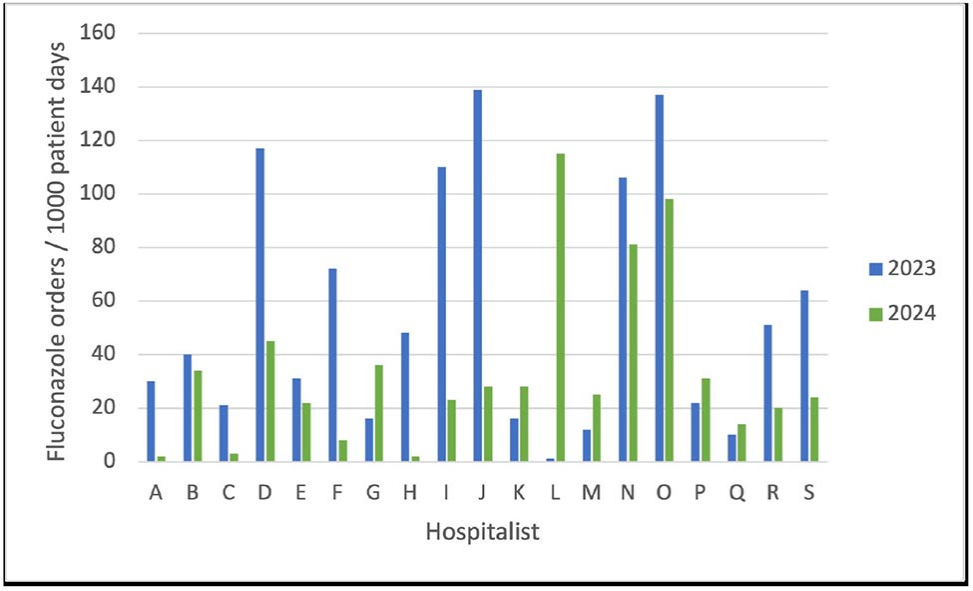
Hospital B - Hospitalist Fluconazole Days of Therapy/1000 Patient Days

**Methods:**

On 1/1/2024 "budding yeast" was removed from urinalysis reports at the two hospitals. Fluconazole prescribing was tracked as days of therapy per 1000 patient days (DOT/1000) for physicians and mid-level prescribers for 1 year pre- and post-intervention. Prescribers with less than 6 months of data except for infectious diseases (ID) were excluded. DOT/1000 days were compared pre- and post- and across various specialties. Specialists with data at both institutions in the health system were combined.

**Results:**

There were 41 providers (12 at hospital A, 21 at hospital B, & 8 at both). Sub-groupings included 4 ID physicians, 3 surgeons, 5 pulmonologists, and 2 nurse practitioners (NP) or physician associates (PA). Comparing hospitalists (12 at A & 16 at B), fluconazole DOT/1000 were reduced from 2023 to 2024 by 37% (427 vs 269) and 54% (895 vs 412) at hospital A & B, respectively (Figures 1 & 2). Ten of 12 hospitalists at hospital A and 12 of 16 at hospital B had decreased DOT/1000 in 2024. For all providers at hospital A, DOT/1000 decreased by 24% in 2024 (894 vs 683), and hospital B decreased by 31% (2191 vs 1517). Sub-groups were combined for both hospitals with NP/PA having a total reduction of 24% (147 vs 112) in 2024, ID physicians decreased by 20% (986 vs 784) and pulmonologists decreased by 9% (456 vs 416). Surgeons increased by 19% (174 vs 207).

**Conclusion:**

After removal of the term “budding yeast” from urinalysis, fluconazole prescribing decreased throughout the health system with the largest impact on hospitalists. Simple, straightforward nudge stewardship interventions can significantly impact prescribing habits.

**Disclosures:**

Christopher M. Bland, PharmD, FCCP, FIDSA, BCPS, Merck: Honoraria|Nestle Health Sciences: Honoraria|Shionogi, Inc.: Advisor/Consultant|Shionogi, Inc.: Honoraria Bruce M. Jones, Pharm.D., FIDSA, BCPS, AbbVie: Advisor/Consultant|AbbVie: Honoraria|Ferring: Grant/Research Support|Ferring: Honoraria|Innoviva: Honoraria|Paratek: Advisor/Consultant|Paratek: Honoraria

